# BioXmark® liquid fiducials to enable radiotherapy tumor boosting in rectal cancer, a feasibility trial

**DOI:** 10.1016/j.ctro.2022.10.013

**Published:** 2022-11-04

**Authors:** Thirza J.S. Opbroek, Yves C.P. Willems, Frank Verhaegen, Rogier de Ridder, Chantal Hoge, Jarno Melenhorst, Frans Bakers, Heike I. Grabsch, Jeroen Buijsen, Evert J. Van Limbergen, Richard A.M. Canters, Maaike Berbée

**Affiliations:** a Department of Radiation Oncology (Maastro), GROW School for Oncology and Developmental Biology, Maastricht University Medical Center+, the Netherlands; b Department of Gastroenterology, Maastricht University Medical Center+, the Netherlands; c Department of Surgery, Maastricht University Medical Center+, the Netherlands; d Department of Radiology, Maastricht University Medical Center+, the Netherlands; e Department of Pathology, Maastricht University Medical Center+, the Netherlands; f Division of Pathology and Data Analytics, Leeds Institute of Medical Research at St James’s, University of Leeds, Leeds, UK

**Keywords:** Liquid fiducial marker, IGRT, Radiotherapy, Rectal cancer

## Abstract

**Background and purpose:**

Dose-escalation in rectal cancer (RCa) may result in an increased complete response rate and thereby enable omission of surgery and organ preservation. In order to implement dose-escalation, it is crucial to develop a technique that allows for accurate image-guided radiotherapy. The aim of the current study was to determine the performance of a novel liquid fiducial marker (BioXmark®) in RCa patients during the radiotherapy course by assessing its positional stability on daily cone-beam CT (CBCT), technical feasibility, visibility on different imaging modalities and safety.

**Materials and methods:**

Prospective, non-randomized, single-arm feasibility trial with inclusion of twenty patients referred for neoadjuvant chemoradiotherapy for locally advanced RCa. Primary study endpoint was positional stability on CBCT. Furthermore, technical aspects, safety and clinical performance of the marker, such as visibility on different imaging modalities, were evaluated.

**Results:**

Seventy-four markers from twenty patients were available for analysis. The marker was stable in 96% of the cases. One marker showed clinically relevant migration, one marker was lost before start of treatment and one marker was lost during treatment. Marker visibility was good on computed tomography (CT) and CBCT, and moderate on electronic portal imaging (*EPI*). Marker visibility on magnetic resonance imaging (MRI) was poor during response evaluation.

**Conclusion:**

The novel liquid fiducial marker demonstrated positional stability. We provide evidence of the feasibility of the novel fiducial marker for image-guided radiotherapy on daily cone beam CT for RCa patients.

## Highlights

• BioXmark® is a novel liquid fiducial marker for image-guided radiotherapy.
• The marker remained stable during chemoradiotherapy in 96% of rectal cancer cases.
• The fiducial allows for image tracking on CT-based imaging modalities.
• Marker visibility was good using CT-based imaging without any relevant artifacts.
• The marker is easy to inject without marker related adverse events.

## Introduction

Neoadjuvant chemoradiotherapy plays an important role in the treatment of patients with locally advanced rectal cancer (RCa). Its effect is two-folded: it induces tumor downsizing and reduces the locoregional recurrence rate [1], [2], [3], [4], [5]. Approximately 15–25 % of patients treated with neoadjuvant chemoradiotherapy achieve a complete response (CR) [6], [7], [8], [9], [10], [11]. Clinical complete responders, in whom no residual viable tumor is left, are potential candidates for the so called “watch and wait” strategy. These patients are closely monitored instead of undergoing surgery. Such a strategy may omit the need for a permanent colostomy and preserve rectal function. From a radiobiological perspective, increasing the radiation dose to the rectal tumor is expected to result in an increased CR rate [12].

To date, results of clinical trials exploring the effect of rectal external beam dose-escalation remain inconclusive [13], [14], [15], [16], [17], [18], [19], [20], [21], [22]. This may be partially due to technical limitations and limited visibility of the tumor on cone beam computed tomography (CT) leading to large planning tumor volume (PTV) margins to correct for rectal displacements, thereby hampering clinically significant dose-escalation. Magnetic resonance (MR) guided radiotherapy is one way to overcome the poor visibility of the rectal tumor. However, to date MR-linac is not widely available. As tumor boost delivery is expected to increase the rate of complete responders, an alternative tumor visualization technique is required to identify the tumor on cone-beam CT (CBCT) imaging modalities prior to each radiotherapy fraction.

A promising solution to this problem is the use of fiducial markers. The use of markers for image-guided radiotherapy in RCa has gained increasing interest in the last couple of years. Currently, three different types of markers are available: surgical clips, lipiodol and gold markers. In 2014 a novel liquid fiducial marker, BioXmark® (Nanovi Radiotherapy A/S, Lyngby, Denmark), became available for clinical research purposes. Hypothesized advantages of the novel marker are increased visibility on different imaging modalities compared to gold markers, easy injection via thin needles, less imaging artifacts and improved positional stability.

The primary objective of this study was to assess the feasibility of using this novel fiducial marker in RCa patients. If proven to be feasible, it may offer a novel tool for adequate tumor localization on CBCT and may thereby allow for use of smaller treatment margins due to less geographic uncertainties. This is the first study to assess the feasibility of the novel marker in RCa patients.

## Materials and methods

### Patient selection

**Fig. 1:**
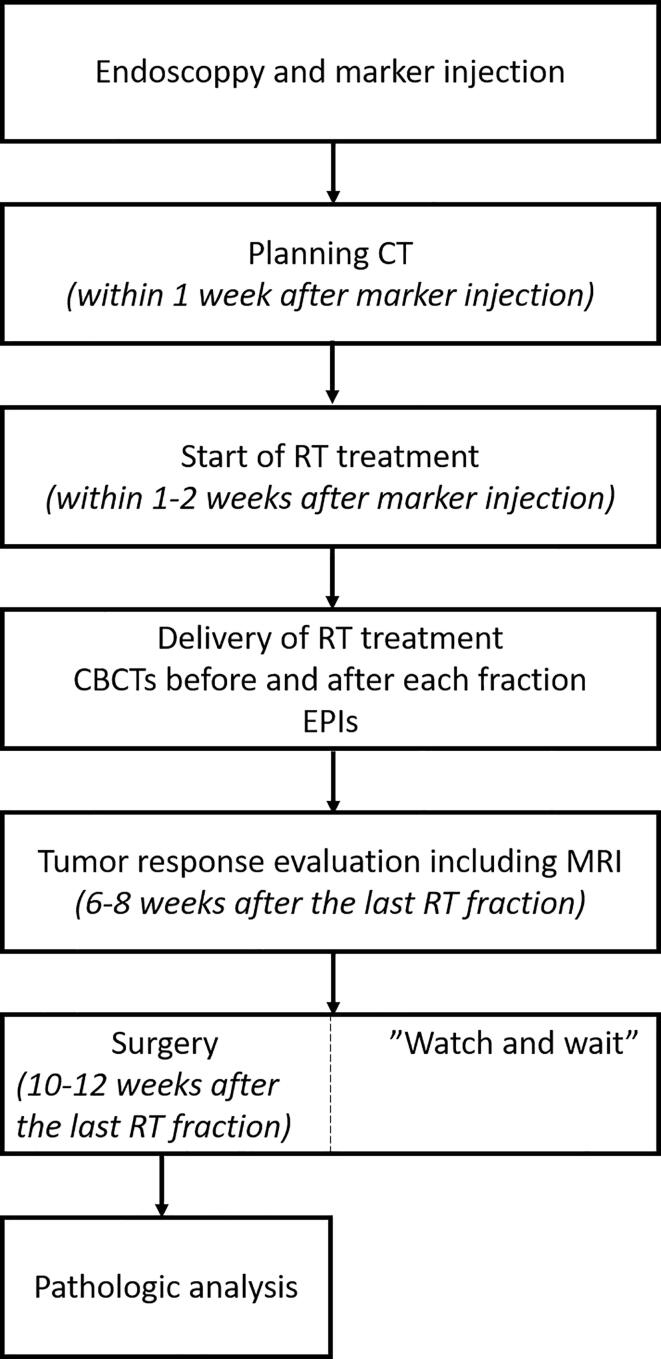
Overview of the various assessments and procedures and their timing relative to marker injection or radiotherapy delivery. *CT = computed tomography; RT = radiotherapy; CBCT = cone-beam computed tomography; EPI = electronic portal imaging; MRI = magnetic resonance imaging*.

This is a prospective, non-randomized, single arm, feasibility trial conducted between January 2018 and February 2019 at Maastro, Maastricht, the Netherlands. The study was approved by the medical ethics committee of the Maastricht University Medical Center+ (MUMC+) (number: METC173018) and was registered on Clinicaltrials.gov (Identifier NCT03265418). Twenty patients were included after written informed consent had been obtained. Inclusion criteria comprised of histological or cytological proven adenocarcinoma of the rectum planned to be treated with neoadjuvant long-course external beam chemoradiotherapy and an age of at least 18 years. Exclusion criteria included a history of an allergic reaction to iodine and anticoagulant usage (platelet aggregation inhibitors or coumarins). All patients received standard treatment for RCa: a total dose of 50 Gray (Gy) in 25 fractions of 2 Gy, delivered in five weeks with concurrent capecitabine two times daily on radiotherapy days. Patients participating in the RECTAL-BOOST trial (NCT01951521), in which patients were randomized to receive no boost or a sequential boost of 15 Gy in five fractions to the gross tumor volume (GTV), were also eligible [22]. Follow-up after completion of chemoradiotherapy was according to standard clinical practice and consisted of tumor response evaluation six to eight weeks after the last radiation fraction. Surgical resection was scheduled eight to twelve weeks after chemoradiotherapy. Clinical complete responders were offered a “watch and wait” strategy according to local protocol. For an overview of the various assessments and procedures, please refer to Fig. 1.

### Fiducial marker

BioXmark® (Nanovi A/S, Lyngby, Denmark), hereinafter referred to as marker, is a novel liquid, biodegradable, non-polymeric, radiopaque injectable fiducial consisting of sucrose acetate isobutyrate (SAIB), electron dense SAIB analogue and ethanol. In contrary to lipiodol which is prone to extravasation into the surrounding tissues and to potential fading or blurring of the spots, the marker is expected to increase its viscosity after injection due to its composition, forming a 3D structure, which further stabilizes the product at the injection site [23], [24], [25]. The marker was injected into the submucosa of the rectal wall after enema preparation during sigmoidoscopy using thin needles (<25 Gauge) by two experienced gastroenterologists. A two-step marker injection method was used to minimize the risk of extra-luminal injection of the marker. First, a saline solution was injected into the submucosal space to create a small bleb of 0.5 mL, followed by marker injection into the bleb. The markers were injected at an angle of approximately 45 degrees to limit risk of perforation. In total four marker spots with a volume of 80 µL each were injected into the rectal wall. Two markers were placed cranially of the tumor and two caudally. Total procedure time was around 15 min.

### Data collection

Patient characteristics were extracted from clinical records and the deidentified patient data were analyzed. The location of the tumor was categorized according to ESMO guidelines; low: up to 5 cm from the anal margin, mid: >5–10 cm from the anal margin, upper: >10–15 cm from the anal margin [26]. Pre-treatment positioning of patients was based on the standard clinical decision protocol. For study purposes, an extra CBCT was obtained after each treatment fraction. In total, patients received 25 extra kilovolt CBCTs. Both the CTs and CBCTs consisted of 3 mm slice thickness.

### Endpoint analysis

All available markers were contoured on CT using the Aria contouring environment (Varian Medical Systems, Version 15.1, Palo Alto, USA) based on a Hounsfield unit (HU) threshold between 400 and the upper limit within a selected volume of interest. Coordinates of the fiducial center were obtained and used as surrogate for marker location. Overlapping markers were considered and delineated as a single marker. Marker pair distances were calculated based on the corresponding coordinates and used to analyze positional stability over the course of treatment by fitting a linear regression model. The corresponding slope of fit was calculated using SPSS and was used as a surrogate for migration. A slope significantly larger than zero was considered as marker migration through the tissue. In such cases, the center of mass of the marker was calculated relative to the other three markers and analyzed with linear regression. If the slope was significantly larger than zero and the total migration distance was larger than 5 mm, it was scored as a potential clinically relevant migration. A slope less than zero was considered to be tumor shrinkage rather than marker migration. The percentage of fiducials lost from injection to CT acquisition and during radiotherapy was calculated based on available coordinates.

Visibility of the fiducials was scored on the planning CT, daily CBCT, electronic portal imaging (*EPI*) and T1, T2 and diffusion weighted MR imaging (MRI) as: 0 = not visible, 1 = barely visible, 2 = clearly visible. Visibility on CT, CBCT and *EPI* was scored by two observers and MRI visibility by one independent radiologist.

The safety of the marker and marker placement was determined based on the occurrence of any related adverse events shortly after marker placement, during radiotherapy and during follow-up until surgery or in case of omission of surgery, until three months after marker placement. This was scored using the Common Terminology Criteria for Adverse Events version 4.0 [27].

Pathologists reviewed the histology of the resection specimens for the presence of tissue alterations which might be attributed to the presence of the fiducials and included this information in the routine pathology report.

### Statistical analysis

Statistical calculations were performed using SPSS (IBM SPSS software version 23). Sample size calculation was based on the positional stability of the markers. The expected sample proportion of migrating markers was set at 8 %. With a two-sided 95 % confidence interval a sample size of 60 markers was necessary [16]. Expecting at least three markers per patient to be available for analysis, twenty patients were required. The cut-off for the primary endpoint was marker stability of at least 85 % of the markers. The mean pair distance, range and standard deviation were calculated. Statistical analysis of the secondary endpoints was performed with descriptive statistics. A p-value of < 0.05 was considered significant.

## Results

Twenty patients undergoing neoadjuvant chemoradiotherapy for RCa were included in this trial, of which nineteen patients were available for analysis. One patient had missing data and was excluded from analysis. Baseline characteristics are summarized in Table 1. Two patients with limited distant metastatic disease were treated with curative intent by neoadjuvant chemoradiotherapy of the RCa and liver metastasectomy. In total, eighty markers were injected in twenty patients. There were no technical problems during the endoscopic injection of the markers.

**Table 1:** Patient characteristics (n = 20). * = both patients with M1 disease had a cTcN stage of cT3N2. *SD = standard deviation, W&W strategy = “watch and wait” strategy, pCR = pathological complete response; RT = radiotherapy*.

Sex (n)	
Male	19
Female	1
Age (mean ± SD; range)	64 ± 9; 48–76
Clinical stage before treatment (n)	
cT2N2	1
cT3N0-2	16
cT4N0-2	1
M1*	2
Pathological stage (n)	
W&W strategy	4
ypT0N0 (pCR)	3
ypT1-2N0-1	6
ypT3 or N2	5
M1 on evaluation	2
Tumor location	
Upper	1
Mid	9
Lower	10
Treatment position	
Supine	20
Average time between consult and start of RT (mean ± SD; range)	12 ± 3.4; 6–18

### Marker stability

**Fig. 2:**
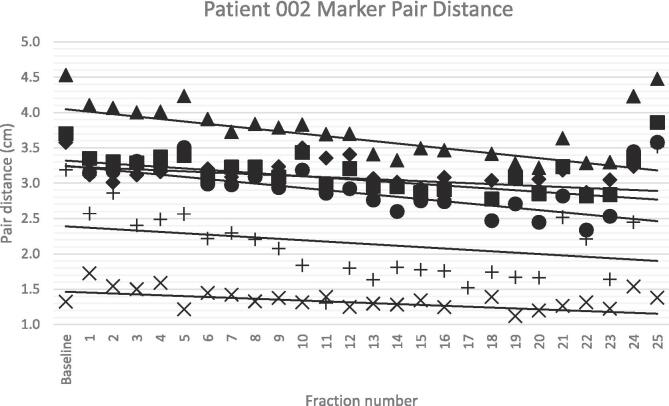
An example of marker pair distances. Daily variation (figures) and linear fit (solid line). Corresponding slope of fit (cm/day): Distance 1–2 (+) −0.043, Distance 1–3 (▲) −0.045, Distance 1–4 (●) −0.041, Distance 2–3 (♦) −0.014, Distance 2–4 (■) −0.025, Distance 3–4 (x) −0.012. Markers have been labeled from cranial to caudal, i.e. marker 1 is the most cranially located marker on CT/CBCT and marker 4 the most caudally located marker.

Eight out of 80 injected markers (10 %) were not available for analysis: two were lost, four markers could not be followed during the entire radiation course due to missing imaging data, and two markers were too close to an adjacent marker and were evaluated as a single marker. This resulted in a total of 106 marker pair distances being available for analysis. Assumptions of linear regression were tested and met in all cases. The mean marker pair distance for all six distances was 4.1 cm (standard deviation (SD): 1.3 cm). Inter-fraction distance showed a large variation with a mean of 1.5 cm and a maximum of 4.2 cm. Individual distances with mean marker pair, mean marker migration and mean total migration are summarized in Table 2. Linear fit through the marker pair distances resulted in an average slope of −0.036 cm per fraction (SD: 0.0062 cm). Total migration per marker pair distance showed an average total migration of −0.5 cm (SD: 0.1 cm). Mean, SD, range and minimum–maximum translation per patient and distance can be found in Appendix A. An example of the daily variation and linear fit of one patient is presented in Fig. 2. Seventy-three out of 106 (69 %) marker pair distances were significantly different from zero, with all but one being negative. Further analysis of the center of mass distance of the marker with the positive slope markers resulted in a significantly larger slope relative to the center of mass with a total migration distance of 4.3 mm, which is lower than the predefined 5 mm margin cut-off. Hence this marker migration was not scored as clinically relevant migration. One marker was found to migrate significantly through the mesorectal fat based on a large variation in day-to-day location. In total, three out of eighty markers (3.8 %) were scored as unstable; two were lost and one migrated.

**Table 2:** Mean marker pair distances, migration per fraction and total migration (slope * fractions) in centimeters (n = 18). Markers have been labeled from cranial to caudal, i.e. marker 1 is the most cranially located marker on CT/CBCT and marker 4 the most caudally located marker. *SD = standard deviation*.

Distance	Mean marker pair distance in cm (SD)	Mean marker migration per fraction (slope) in cm	Mean total migration (cm)
1–2	2.67 (0.39)	−0.01	−0.29
1–3	5.26 (0.41)	−0.02	−0.55
1–4	4.74 (0.38)	−0.02	−0.53
2–3	4.93 (0.40)	−0.02	−0.62
2–4	4.59 (0.36)	−0.02	−0.59
3–4	2.28 (0.31)	−0.02	−0.52

### Visibility

All markers still in situ were clearly visible on the planning CT-scan and 98.5 % of the markers were clearly visible on daily CBCTs. Minor beam hardening artifacts were present on CBCT without significantly impacting image interpretation. EPIs were available for analysis in eleven patients; in the other eight patients the EPIs could not be retrieved for analysis due to technical errors. Visibility on *EPI* varied with mostly only moderate visibility. As the rectum is located ventral to the bony structures of the sacrum and hip, marker visibility on *EPI* is dependent on the location of the markers and the bone density in the background. This resulted in four cases (36 %) in which the markers could not be clearly differentiated on *EPI*. Examples of the marker on various imaging modalities are included in Appendix B.

Six to eight weeks after completion of chemoradiotherapy an MRI was performed for tumor response evaluation purposes. Marker visibility was assessed by one independent radiologist on T1, T2 and diffusion weighted sequences with 3 mm slice thickness. None of the markers were visible.

### Adverse events

One patient experienced a vagal reaction during marker injection with spontaneous recovery and no late side effects. In three patients, technical difficulties with marker injection were experienced with possible leakage of the fiducial into the lumen of the rectum in two patients. In one case the pressure in the needle was potentially increased due to a second aspiration of the liquid solution. No adverse events were reported shortly after injection or during radiotherapy in any of the patients. At two months follow-up, the only treatment-related acute toxicities were Grade 1 proctitis in 7/20 patients (35 %) and Grade 2 proctitis in 1/20 patients (5 %), scored according to the Common Terminology Criteria for Adverse Events Common Terminology Criteria for Adverse Events (CTCAE) version 4.03 [27].

### Pathology reporting

Sixteen resection specimens were available for evaluation. One case suggested marker related inflammation by the presence of multinucleated giant cells located around foreign material presumed to be the fiducial.

## Discussion

This is the first study to investigate the feasibility of this novel liquid fiducial marker as a tumor location surrogate on daily CBCT in RCa patients. In order to be feasible for clinical usage, the marker should not migrate or disappear, should not cause any adverse events and should be clearly visible on different imaging modalities. Fiducial markers can generally provide information regarding inter- and intra-fractional tumor position during radiotherapy. Jin *et al*. provided insight into the position variation of esophageal tumors with the use of different types of markers [28]. Van der Horst *et al*. did the same in pancreatic tumors [29]. In vivo measurements of the stability of the marker over a 5-month period showed movement of less than 2 mm [30]. Hence, fiducial markers may provide a potential benefit in gastrointestinal tumors to allow for precise day-to-day position verification. With a stable, clearly visible and easily injected liquid marker, the inter- and intra-fractional tumor movement and the correspondingly required PTV margins can be determined.

The novel fiducial marker demonstrated good positional stability during neoadjuvant chemoradiotherapy. Analysis of the plotted slopes of linear fits did show that the markers tended to move towards each other. As chemoradiotherapy for RCa is known to induce substantial tumor shrinkage, this movement was interpreted as tumor shrinkage rather than marker migration as prespecified in the protocol [31], [32], [33], [34], [35], [36], [37]. In addition, the large variation in inter-fraction pair distances is most likely explained by day-to-day variation in rectum and bladder filling. Furthermore, it is important to note that the use of a surrogate for marker migration might result in an underestimation of the true migration, due to potential simultaneous migration in the same direction of marker pairs. Moreover, sex-based differences might be a potential weakness of this study; sexual intercourse might be more likely to cause marker displacement in female patients than in male patients.

Van den Ende *et al*. tested four different types of fiducials including visicoil and gold markers for stability and MRI visibility [38]. The overall stability was poor since only 39 out of 64 injected fiducials were available on the first imaging modality. After one week of radiotherapy, only 35 markers were still in situ. The majority of the markers were lost between insertion and the first MRI (n = 18), some were injected into the prostate (n = 5) and a few were lost in the first week of radiotherapy (n = 4) [38]. These results exemplify the limitations of frequently used fiducials and emphasize the need for a stable fiducial marker, especially in RCa patients.

Visibility of the novel marker on CT and CBCT was good with only minor problems regarding formation of imaging artifacts on CBCT. Several other studies have evaluated visibility of the liquid fiducial on different imaging modalities and in other tumor sites. Evaluation of visibility by De Roover *et al*. using different volumes of the marker on phantom models in comparison to gold fiducials showed less artifacts on CT and CBCT in favor of the marker, especially when using volumes less than 100 μL [39]. Rydhög *et al*. analyzed 29 markers in 15 patients with locally advanced lung cancer and provided evidence that the markers were well visible on CBCT [40]. Likewise, De Ridder *et al*. noted that all visible markers after CT acquisition were still clearly visible on the last CBCT, and that there was no significant migration of markers [24]. These results are in line with our results on marker visibility on CBCT and the current results also demonstrate proper marker stability. Moreover, an in vivo study showed a significant advantage of the marker over gold and polymer fiducials with regards to imaging artifacts on CT imaging [26]. According to previous evaluation, the liquid marker should be hypo-intense on both T1 and T2-weighted MRI [41]. However, in the current study the fiducials were not visible on post-treatment MRI. This may be due to the low volume of the markers combined with a 3 mm slice thickness. Machiels *et al.* mentioned that for esophageal cancer the markers were invisible on T2-weighted MRI, but that the marker became visible with a higher in-plane resolution [41]. They recommended a slice thickness of < 3 mm and an in-plane resolution of at least 1x1 mm, and for volumes < 0.1 mL, such as in this study, they note that an even higher in-plane resolution was required for good visibility [41]. Additionally, there are many hypo-intense structures visible on MRI in and around the rectum and bowel bag, which makes it hard to differentiate a hypo-intense marker. Furthermore, in the current study MRI was only evaluated at a single point in time (approximately 12–15 weeks after marker injection) and the MRI sequences were not optimized specifically for detection of the marker. More research is needed to investigate the potential role of this fiducial in MR-guided radiotherapy. A solution may be adding a contrast agent such as gadolinium into the marker.

In the current study, no injection or marker related acute or late toxicity was encountered during follow-up. Moreover, pathology did not report any substantial local tissue inflammation. A study by De Blanck *et al*. reports on long-term safety of the fiducial with a median follow-up of 34 months. In this study, the marker was injected into the tumor and lymph nodes of NSCLC patients. No major safety or toxicity issues were observed [42]. The markers remained in situ and were not completely degraded at 36 months.

Limitations of the present study include uncertainties in distance between marker and tumor location, and the lack of a gold standard to verify the tumor location since marker stability was evaluated based on a surrogate (relative marker positioning). Although the location was chosen with maximum care by the gastroenterologists, being approximately 1 cm from the outer tumor border, this may vary. Furthermore, reproducibility of our injection method needs external validation as it may be operator dependent. Ideally, this novel marker would have been directly compared to the performance of other markers. Strengths include standardized delineation of the individual markers and analyses based on marker pair distance. Sample size was calculated beforehand to ensure statistical power.

In conclusion, we provide evidence of the feasibility of the novel fiducial marker, BioXmark®, for image-guided radiotherapy on daily CBCT for RCa patients. The marker may provide a tool for accurate determination of the day-to-day tumor location and thereby allow for safe dose-escalation.

## Declaration of Competing Interest

The authors declare the following financial interests/personal relationships which may be considered as potential competing interests: Authors Berbée, Verhaegen and Van Limbergen hold a patent for a rectal brachytherapy applicator: “Endorectal probe device for effecting radiation treatment of colorectal cancerous tissue in the rectum of a human or animal subject.”, filed December 2016, F. Verhaegen, M. Bellezzo, E. van Limbergen, M. Berbée, B. Reniers, G. Fonseca, EP16204735. The patent has been licensed to Varian Medical Systems, Inc. The conduction of the trial was funded by Nanovi and the BioXmark® fiducials were provided without costs. Nanovi was not involved in the study design, collection, analysis and interpretation of data, writing of the manuscript or the decision to submit the manuscript for publication.
