# Supplementary material for: BioXmark® liquid fiducials to enable radiotherapy tumor boosting in rectal cancer, a feasibility trial
Source: Clin Transl Radiat Oncol. 2022 Nov 4;38:90–5. doi: 10.1016/j.ctro.2022.10.013 (PMC9668658; doi:10.1016/j.ctro.2022.10.013)
Supplement: Supplementary data 1 [file mmc1.docx]

**Table A.1 (Appendix A.1).** Marker distance: mean, SD, min-max, range. Markers have been labeled from cranial to caudal, i.e. marker 1 is the most cranially located marker on CT/CBCT and marker 4 the most caudally located marker.

| **Patient ID and marker distance** | **Mean (cm)** | **SD (cm)** | **Min-max (cm)** | **Range (cm)** |
| --- | --- | --- | --- | --- |
| 001-distance12 | 7.6557 | 1.1840 | 5.86-9.40 | 3.5400 |
| 001-distance13 | 6.9277 | 1.0212 | 5.41-8.56 | 3.1500 |
| 001-distance14 | 9.6833 | 1.1462 | 8.13-11.61 | 3.4800 |
| 001-distance23 | 2.4866 | 0.1609 | 2.15-2.76 | 0.6100 |
| 001-distance24 | 1.8584 | 0.3451 | 1.15-2.50 | 1.3500 |
| 001-distance34 | 3.4356 | 0.2558 | 3.02-3.88 | 0.8600 |
|  |  |  |  |  |
| 002-distance12 | 2.1138 | 0.4745 | 1.31-3.19 | 1.8800 |
| 002-distance13 | 3.7571 | 0.3780 | 3.22-4.53 | 1.3100 |
| 002-distance14 | 2.9585 | 0.3518 | 2.34-3.67 | 1.3300 |
| 002-distance23 | 3.1928 | 0.1930 | 2.87-3.58 | 0.7100 |
| 002-distance24 | 3.1512 | 0.2427 | 2.78-3.70 | 0.9200 |
| 002-distance34 | 1.3626 | 0.1392 | 1.12-1.72 | 0.6000 |
|  |  |  |  |  |
| 004-distance13 | 4.6721 | 0.1795 | 4.12-4.87 | 0.7500 |
| 004-distance14 | 4.7090 | 0.1878 | 4.27-5.08 | 0.8100 |
| 004-distance34 | 2.0823 | 0.1989 | 1.79-2.53 | 0.7400 |
|  |  |  |  |  |
| 005-distance12 | 3.0007 | 0.5787 | 2.15-4.53 | 2.3800 |
| 005-distance13 | 6.1688 | 0.5173 | 5.29-7.48 | 2.1900 |
| 005-distance14 | 6.0975 | 0.2994 | 5.52-6.79 | 1.2700 |
| 005-distance23 | 7.3081 | 0.4694 | 6.60-8.37 | 1.7700 |
| 005-distance24 | 6.4821 | 0.4443 | 5.22-7.15 | 1.9300 |
| 005-distance34 | 3.1690 | 0.4444 | 2.31-4.23 | 1.9200 |
|  |  |  |  |  |
| 006-distance12 | 1.4929 | 0.3492 | 1.07-2.35 | 1.2800 |
| 006-distance13 | 5.6383 | 0.4711 | 4.93-6.70 | 1.7700 |
| 006-distance14 | 5.0580 | 0.5407 | 4.34-6.13 | 1.7900 |
| 006-distance23 | 6.0406 | 0.5795 | 5.35-7.49 | 1.1400 |
| 006-distance24 | 4.9571 | 0.4943 | 4.16-6.02 | 1.8600 |
| 006-distance34 | 2.1964 | 0.7277 | 1.36-4.41 | 3.0500 |
|  |  |  |  |  |
| 007-distance12 | 2.1196 | 0.7265 | 1.19-3.91 | 2.7200 |
| 007-distance13 | 4.9028 | 0.6357 | 4.08-6.70 | 2.6200 |
| 007-distance14 | 4.6146 | 0.5949 | 3.50-6.00 | 2.5000 |
| 007-distance23 | 4.6904 | 0.5012 | 4.02-5.72 | 1.7000 |
| 007-distance24 | 5.4144 | 0.8219 | 4.01-7.11 | 3.1000 |
| 007-distance34 | 2.4913 | 0.5395 | 1.69-3.74 | 2.0500 |
|  |  |  |  |  |
| 008-distance12 | 5.3280 | 0.2370 | 4.66-5.96 | 1.3000 |
| 008-distance13 | 6.0527 | 0.3925 | 5.37-6.93 | 1.5600 |
| 008-distance14 | 5.2346 | 0.3870 | 4.63-6.30 | 1.6700 |
| 008-distance23 | 3.2789 | 0.5411 | 2.60-4.93 | 2.3300 |
| 008-distance24 | 5.1256 | 0.4554 | 4.42-6.40 | 1.9800 |
| 008-distance34 | 2.7862 | 0.2867 | 2.31-3.56 | 1.2500 |
|  |  |  |  |  |
| 010-distance12 | 2.7583 | 0.4186 | 2.19-3.70 | 1.5100 |
| 010-distance13 | 6.4494 | 0.3612 | 5.54-6.96 | 1.4200 |
| 010-distance14 | 5.7237 | 0.3662 | 4.98-6.35 | 1.3700 |
| 010-distance23 | 5.6999 | 0.3211 | 4.97-6.61 | 1.6400 |
| 010-distance24 | 5.5820 | 0.1731 | 5.26-5.89 | 0.6300 |
| 010-distance34 | 1.4200 | 0.1330 | 1.16-1.74 | 0.5800 |
|  |  |  |  |  |
| 011-distance12 | 2.7163 | 0.4092 | 2.18-3.65 | 1.4700 |
| 011-distance13 | 8.3010 | 0.6801 | 7.01-9.48 | 2.4700 |
| 011-distance14 | 7.6118 | 0.4519 | 6.86-8.52 | 1.6600 |
| 011-distance23 | 8.8749 | 1.0588 | 6.73-10.90 | 4.1700 |
| 011-distance24 | 7.6326 | 0.7797 | 6.14-9.55 | 3.4100 |
| 011-distance34 | 2.4641 | 0.7865 | 1.57-4.70 | 3.1300 |
|  |  |  |  |  |
| 012-distance12 | 2.1777 | 0.2165 | 1.74-2.79 | 1.0500 |
| 012-distance13 | 3.6871 | 0.2567 | 2.97-4.16 | 1.1900 |
| 012-distance14 | 4.3937 | 0.3165 | 3.60-4.96 | 1.3600 |
| 012-distance23 | 5.3716 | 0.1750 | 5.05-5.72 | 0.6700 |
| 012-distance24 | 5.6451 | 0.3141 | 5.02-6.32 | 1.3000 |
| 012-distance34 | 2.8614 | 0.2798 | 2.40-3.64 | 1.2400 |
|  |  |  |  |  |
| 013-distance12 | 1.9167 | 0.2650 | 1.47-2.53 | 1.0600 |
| 013-distance13 | 3.8607 | 0.1103 | 3.66-4.09 | 0.4300 |
| 013-distance14 | 4.7875 | 0.2322 | 4.39-5.33 | 0.9400 |
| 013-distance23 | 3.3302 | 0.2797 | 2.80-4.15 | 1.3500 |
| 013-distance24 | 4.7326 | 0.3037 | 4.31-5.52 | 1.2100 |
| 013-distance34 | 1.6623 | 0.1507 | 1.46-2.05 | 0.5900 |
|  |  |  |  |  |
| 014-distance12 | 1.5447 | 0.4045 | 1.05-2.66 | 1.6100 |
| 014-distance134 | 5.8017 | 0.2266 | 5.37-6.18 | 0.8100 |
| 014-distance234 | 4.9938 | 0.3462 | 3.96-5.72 | 1.7600 |
|  |  |  |  |  |
| 015-distance12 | 0.7713 | 0.0755 | 0.65-0.94 | 0.2900 |
| 015-distance13 | 6.2128 | 0.2350 | 5.70-6.63 | 0.9300 |
| 015-distance14 | 5.2488 | 0.2548 | 4.76-5.66 | 0.9000 |
| 015-distance23 | 6.4901 | 0.2324 | 5.86-6.88 | 1.0200 |
| 015-distance24 | 5.3688 | 0.2446 | 4.75-5.86 | 1.1100 |
| 015-distance34 | 1.7746 | 0.0926 | 1.60-1.96 | 0.3600 |
|  |  |  |  |  |
| 016-distance12 | 1.3543 | 0.4272 | 0.55-2.26 | 1.7100 |
| 016-distance13 | 5.0091 | 0.4167 | 4.45-6.10 | 1.6500 |
| 016-distance14 | 2.8719 | 0.3808 | 2.37-3.80 | 1.4300 |
| 016-distance23 | 5.1580 | 0.4458 | 4.16-6.34 | 2.1800 |
| 016-distance24 | 2.1339 | 0.2797 | 1.77-2.73 | 0.9600 |
| 016-distance34 | 3.6539 | 0.3926 | 3.14-4.95 | 1.8100 |
|  |  |  |  |  |
| 017-distance12 | 1.8568 | 0.2163 | 1.59-2.31 | 0.7200 |
| 017-distance13 | 4.7276 | 0.2194 | 4.34-5.15 | 0.8100 |
| 017-distance14 | 4.1919 | 0.1451 | 3.97-4.43 | 0.4600 |
| 017-distance23 | 3.9091 | 0.2791 | 3.41-4.39 | 0.9800 |
| 017-distance24 | 2.8493 | 0.2348 | 2.45-3.26 | 0.8100 |
| 017-distance34 | 1.6170 | 0.1717 | 1.34-2.14 | 0.8000 |
|  |  |  |  |  |
| 018-distance12 | 4.9435 | 0.4511 | 6.15-4.22 | 1.9300 |
| 018-distance14 | 2.2353 | 0.5792 | 3.59-0.87 | 2.3400 |
| 018-distance24 | 5.2614 | 0.4025 | 5.99-4.63 | 1.1600 |
| 018-marker3 |  |  |  |  |
|  |  |  |  |  |
| 019-distance12 | 1.8313 | 0.1705 | 1.55-2.44 | 0.8900 |
| 019-distance13 | 3.0401 | 0.3948 | 1.79-3.76 | 1.9700 |
| 019-distance14 | 1.5281 | 0.1439 | 1.29-1.83 | 0.5400 |
| 019-distance23 | 3.4418 | 0.4231 | 2.21-4.25 | 2.0400 |
| 019-distance24 | 2.8115 | 0.1553 | 2.51-3.15 | 0.6400 |
| 019-distance34 | 1.9106 | 0.1766 | 1.54-2.28 | 0.7400 |
|  |  |  |  |  |
| 020-distance12 | 1.8313 | 0.1705 | 1.55-2.44 | 0.8900 |
| 020-distance13 | 3.7600 | 0.3948 | 1.79-3.76 | 1.9700 |
| 020-distance14 | 1.8300 | 0.1439 | 1.29-1.83 | -0.5400 |
| 020-distance23 | 4.2500 | 0.4231 | 2.21-4.25 | 2.0400 |
| 020-distance24 | 3.1500 | 0.1553 | 2.51-3.15 | 0.6400 |
| 020-distance34 | 2.2800 | 0.1766 | 1.54-2.28 | 0.7400 |
|  |  |  |  |  |
| 022-distance12 | 2.6107 | 0.2186 | 2.24-3.12 | 0.8800 |
| 022-distance13 | 5.6872 | 0.4671 | 4.97-6.58 | 1.6100 |
| 022-distance14 | 5.4972 | 0.3751 | 4.80-6.09 | 1.3000 |
| 022-distance23 | 5.3601 | 0.3870 | 4.50-6.09 | 1.5800 |
| 022-distance24 | 5.4201 | 0.3364 | 4.83-6.05 | 1.2200 |
| 022-distance34 | 1.5587 | 0.2327 | 1.06-1.95 | 0.8900 |


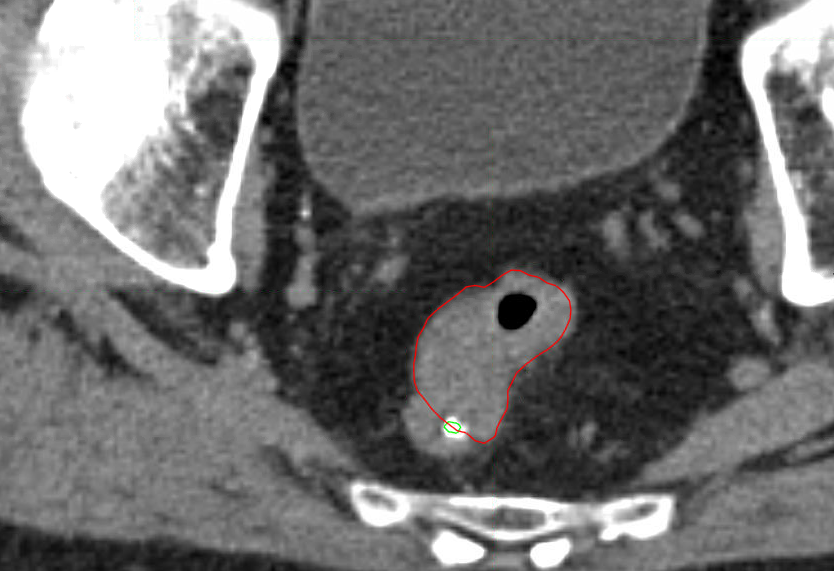


**Figure B.1 (Appendix B.1)**. Planning CT (window width 400; window level 40; slice thickness 3 mm) with a clearly visible hyperintense marker. Contouring: green = marker; red = gross tumor volume (GTV).


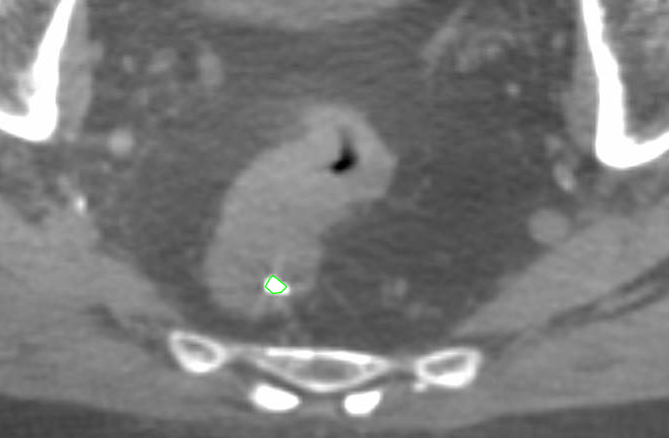


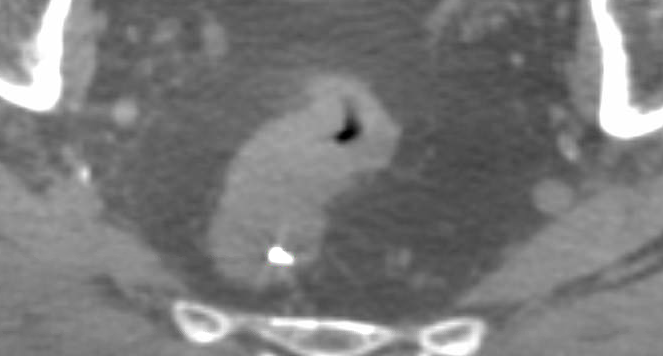


**Figure B.2 (Appendix B.2)**. CBCT (slice thickness: 3mm) after the first fraction displaying the corresponding hyperintense marker. Minor (beam hardening) imaging artifacts can be noticed. Top: including contouring (green = marker). Bottom: excluding contouring to improve visualization of imaging artifacts.


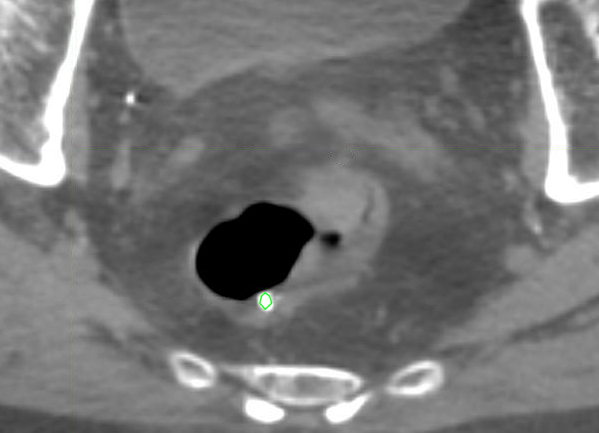


**Figure B.3 (Appendix B.3)**. CBCT (slice thickness: 3mm) after the twentieth fraction displaying the corresponding hyperintense marker. Contouring: green = marker. Imaging artifacts are significantly reduced compared to Figure B.3.
